# Clustered Regularly Interspaced Short Palindromic Repeats/CRISPR-Associated Protein 9 Mediated Knockout Reveals Functions of the *yellow-y* Gene in *Spodoptera litura*

**DOI:** 10.3389/fphys.2020.615391

**Published:** 2020-12-17

**Authors:** Xiao-Long Liu, Wei-Kang Han, Long-Ji Ze, Ying-Chuan Peng, Yi-Lin Yang, Jin Zhang, Qi Yan, Shuang-Lin Dong

**Affiliations:** ^1^ Key Laboratory of Integrated Management of Crop Disease and Pests, Ministry of Education/Department of Entomology, Nanjing Agricultural University, Nanjing, China; ^2^ Institute of Entomology, Jiangxi Agricultural University, Nanchang, China; ^3^ Department of Evolutionary Neuroethology, Max Planck Institute for Chemical Ecology, Jena, Germany

**Keywords:** CRISPR/Cas9, Spodoptera litura, pigmentation, molting, yellow-y

## Abstract

*Yellow* genes are thought to be involved in the melanin biosynthetic pathway and play a crucial role in pigmentation reactions in insects. However, little research has been done on *yellow* genes in lepidopteran pests. To clarify the function of one of the *yellow* genes (*yellow-y*) in *Spodoptera litura*, we cloned the full-length of *yellow-y*, and investigated its spatial and temporal expression profiles by quantitative real-time PCR (qPCR). It revealed that *yellow-y* was highly expressed in larva of fourth, fifth, and sixth instars, as well as in epidermis (Ep), fat bodies (FB), Malpighian tubes (MT), and midguts (MG) of the larvae; whereas it was expressed in very low levels in different tissues of adults, and was almost undetected in pupa. This expression profile suggests an important role of *yellow-y* in larvae, minor role in adults, and no role in pupae. To confirm this, we disrupted *yellow-y* using the clustered regularly interspaced short palindromic repeats/CRISPR-associated protein 9 (CRISPR/Cas9) system, and obtained G0 insects with mutation in *yellow-y*. The mutation in *yellow-y* clearly rendered the larvae body, a color yellower than that of wide type insects, and in addition, the mutation resulted in abnormal segmentation and molting for older larvae. The mutation of *yellow-y* also made various adult tissues (antennae, proboscis, legs, and wings) yellowish. However, the mutation had no effect on pigmentation of the pupal cuticle. Taken together, our study clearly demonstrated the role of *yellow-y* not only in the body pigmentation of larvae and adults, and but also in segmentation and molting of larvae, providing new insights into the physiology of larval development, as well as a useful marker gene for genome editing based studies.

## Introduction

Coloration is deeply related to the adaptation and survival of insects, as it is involved in cuticle formation, crypsis, mimicry, aposematism, mating signals, and others (Tsuchida et al., 2010). Cuticle pigmentation contributes to forming a variety of insect coloration. Melanin is the main protective pigment found in insect cuticles (Shamim et al., 2014), which functions as a shield against damaging UV light and serves as a defense mechanism to encapsulate foreign organisms (Hearing and Tsukamoto, 1991; Sugumaran, 2002; d’Ischia et al., 2013). Melanin is also the major cuticular pigment and plays an important role in the darkening and hardening of insect cuticles (Hopkins and Kramer, 1992; Arakane et al., 2009; Andersen, 2010).

The *yellow* genes are thought to be involved in the biosynthesis pathway of melanin in insects. The first characterized gene of this family was named *yellow* in *Drosophila melanogaster* (Nash and Yarkin, 1974). In *Drosophila*, the mutant of *yellow* gene not only affects the morphology, such as producing mosaics pattern in some cuticle structures, but also affects the behavior, such as decrement in locomotor activity or competitive mating ability in male (Dow, 1976; Wilson et al., 1976; Burnet and Wilson, 1980; Inestrosa et al., 1996; d’Ischia et al., 2013). In *Tribolium castaneum*, double-stranded RNA (dsRNA)-mediated transcript knockdown revealed that different yellow genes play different roles: *yellow-y* is required for melanin production in the hindwings, *yellow-f* is necessary for adult cuticle sclerotization but not for pigmentation (Arakane et al., 2010), and *yellow-e* gene is involved in body coloration and anti-dehydration (Noh et al., 2015). In another coleopteran insect *Tenebrio molitor*, knockdown of *yellow-y* gene by RNA interference (RNAi) leads to decrease of black pigmentation in the middle mesocuticle layer and hindwing (Mun et al., 2020). Considering the inefficiency of RNAi in lepidopteran insect species (Wang et al., 2016; Peng et al., 2020), a more effective gene knockdown or knockout technology is essential for the functional studies of yellow genes.

The clustered regularly interspaced short palindromic repeats/CRISPR-associated protein 9 (CRISPR/Cas9) system is a newly developed and an effective genome editing technology, which has been used in gene function study in some lepidopterans, such as *Helicoverpa armigera* (Chang et al., 2017; Ye et al., 2017; Wang et al., 2018), *Plutella xylostella* (Liu et al., 2020), and *Manduca sexta* (Fandino et al., 2019). By using CRISPR/Cas9 system, the *yellow-y* gene has been recently identified to be involved in synthesizing melanin in *Bicyclus anynana* (Matsuoka and Monteiro, 2018), *Agrotis ipsilon* (Chen et al., 2018), and *P. xylostella* (Wang et al., 2020), but phenotypes (observable morphological, developmental, and behavioral properties) induced by the knockout of *yellow-y* were not very consistent among these species. Thus, more studies are undoubtedly needed to further refine the link between the phenotypes and the yellow genes.

The tobacco cutworm, *Spodoptera litura* (*Lepidoptera*, *Noctuidae*) is one of the most devastating agricultural pests worldwide (Wei et al., 2004; Cheng et al., 2017). In the current work, 11 yellow genes from *S. litura* were cloned according to the sequences in NCBI database. Then, the spatial and temporal expression pattern of *yellow-y* was analyzed by quantitative real-time PCR (qPCR). Finally, the functions of *yellow-y* in cuticle pigmentation and larval development were clarified by knocking-out the genes using CRISPR/Cas9 system.

## Materials and Methods

### Insect Rearing and Tissue Collection

The *S. litura* larvae were reared in the laboratory on an artificial diet at 26 ± 1°C with a 14 h light: 10 h dark photoperiod, and 65 ± 5% with relative humidity (Huang et al., 2002). Pupae were sexed and kept in separate cages. After eclosion, moths were fed with 10% honey solution.

The adult tissues (90 antennae, 90 proboscis, 90 labipalps, 60 heads, 30 thoraces, 30 abdomens, 60 legs, and 60 wings) were dissected and collected from the 1-day-old adults. The larval tissues [silk glands, hemolymphs, Malpighian tubules, fat bodies, and midguts (MG)] were dissected and collected from 90 larvae of sixth instar. In addition, whole insects at different developmental stages were collected, including 300 eggs, 300 first, 300 second, 150 third, 90 fourth, 60 fifth, and 60 sixth instar larvae, 60 male and 60 female pupae (FP), respectively. After collection, the samples were immediately frozen in liquid nitrogen and stored at −80°C for use.

### RNA Isolation and cDNA Synthesis

Total RNA was extracted from samples collected from different developmental stages and different tissues using Trizol Reagent (Invitrogen, Carlsbad, United States) according to the manufacturer’s instructions. The quantity of RNA was determined with NanoDrop-2000 (Thermo Scientific, Waltham, MA, United States). The first single strand complementary DNAs (cDNAs) were synthesized from 1 μg total RNA using HiScript III RT SuperMix for qPCR (+gDNA wiper; Vazyme, Nanjing, China) following the provided protocol.

### Cloning and Analysis of Yellow Genes

The 11 genes encoding yellow proteins were obtained from NCBI, and the complete open reading frame (ORF) of each gene was predicted using the ORFfinder.^1^ To verify the full-length sequence of *yellow-y* gene, the specific primers (Supplementary Table S1) were designed according to cDNA sequence. The PCR reaction was performed in 25 μl containing 12.5 μl of 2 × Phanta Max Master Mix, 9.5 μl of ddH_2_O, 1 μl of cDNA template, and 1 μl of each primer (10 μM). PCR program was carried out under the following conditions: 95°C for 3 min; 35 cycles of 95°C for 15 s, 55°C for 15 s, 72°C for 1 min 30 s; 72°C for 5 min. PCR product was run on a 1.2% agarose gel and the band was recovered and purified by AxyPrep™ DNA Gel Extraction Kit (Axygen, Suzhou, China). Purified PCR product was subcloned into *pEASY*-Blunt vector (TransGen Biotech, Beijing, China) for sequencing. The major royal jelly proteins (MJRPs) domain of each yellow gene was predicted with InterPro.^2^ The genomic sequence was obtained from genome data (Cheng et al., 2017), and the extron-intron structure was determined by comparison of genomic and cDNA sequences.

### Phylogenetic Analysis

Multiple sequence alignments of full-length Yellow proteins from *S. litura*, *Bombyx mori* (Xia et al., 2006), *T. castaneum* (Arakane et al., 2010), *D. melanogaster* (Drapeau, 2001), and *A. ipsilon* (Chen et al., 2018) were performed in ClustalW (Thompson et al., 1994). The phylogenetic tree was constructed using the neighbor-joining method in MEGA6.0 with a bootstrap of 1,000 replicates (Tamura et al., 2013).

### Quantitative Real-Time PCR

To determine the stage and tissue expression profiles of *yellow-y* gene, RNA templates were prepared from different developmental stages and different adult and larva tissues. The specific primers for qPCR were designed using Beacon Designer 7.0 (PRIMER Biosoft International, CA, United States; Supplementary Table S1). The qPCR was performed on QuantStudio™ 6 Flex Real-Time PCR System (Applied Biosystems, Foster City, CA, United States) with ChamQ™ Universal SYBR® qPCR Master Mix (Vazyme, Nanjing, China) according to the manufacturer’s instructions. Each 20 μl reaction solution containing 10 μl of 2 × ChamQ Universal SYBR qPCR Master Mix, 0.4 μl of each primer (10 μM), 2 μl of cDNA template, and 7.2 μl of nuclease-free water. The cycling parameters were: 95°C for 30 s, 40 cycles of 95°C for 5 s, and 60°C for 34 s. The glyceraldehyde-3-phosphate dehydrogenase (*GAPDH*) and elongation factor 1*α* (*EF1α*) were used as references to normalize the target gene expression (Liu et al., 2019). Each reaction was run in three technical replicates, and the experiment was repeated three times using three independent RNA samples. The relative expression levels of *yellow-y* were calculated using the 2^−*Δ*ΔCT^ method (Livak and Schmittgen, 2001).

### Preparation of sgRNA and Cas9 Protein

According to the criteria: 5'-GG-(N) 18-NGG-3' (underlined is the PAM sequence), a 23 bp small guide RNA (sgRNA) targeting site (5'-CCTTTGGATGCCCCCTACGATCC-3') was identified in exon 2 of *yellow-y* genomic NDA sequence (Figure 3A). The sgRNA template was synthesized with Mix Precision gRNA Synthesis Kit (Ambion, Foster City, CA, United States) according to the manufacturer’s instruction. The synthesized sgRNA was purified with gRNA Clean Up Kit (Ambion, Foster City, CA, United States) following the manufacturer’s instructions. The Cas9 protein (TrueCut™ Cas9 Protein v2) was purchased from Thermo Fisher Scientific (Shanghai, China).

### Embryo Microinjection

The collection and preparation of eggs were carried out as previous study (Zhu et al., 2017). Briefly, fresh eggs laid within 2 h were washed down and soaked in 1% methanal solution for 5 min, and then the eggs were lined up on a microscope slide. About 1 nl mix of sgRNA (350 ng/μl) and Cas9 protein (200 ng/μl) were injected into individual eggs using FemtoJet and InjectMan NI 2 microinjection system (Eppendorf, Hamburg, Germany). Injected eggs were incubated at 26 ± 1°C for hatching.

### Mutation Detection and Phenotype Screening

To detect the mutations, 20 injected eggs were collected 24 h after injection and subject to extract the genomic DNA using QIAamp DNA Mini Kit (Qiagen, Hilden, Germany). The fragment (794 bp) including the CRISPR target site was amplified using a pair of specific primers (forward: 5'-AATTTGAAGTACACGGGTACCA-3', reverse: 5'-CGTTCTTATGTTATTAAGCTATGCT-3'). The PCR products were purified using AxyPrep PCP Cleanup Kit (Axygen, Suzhou, Jiangsu, China). The restriction enzyme T7 endonuclease I (New England Biolabs, Ipswich, MA, United States) was used to determine the potential mutations.

Hatched larvae were collected and fed with an artificial diet. The mutant phenotype of larvae, pupae, and adults were checked and imaged digitally under a Nikon SMZ 25 stereo microscopy (Nikon, Japan). The images were converted to RGB stack images, and color intensity (darkness of pigment) in regions of the forewing and veins of the hindwing was measured as mean gray scale values using ImageJ software.^3^

## Results

### Cloning and Phylogenetic Analysis of *Spodoptera litura* Yellow Genes

**Figure 1:**
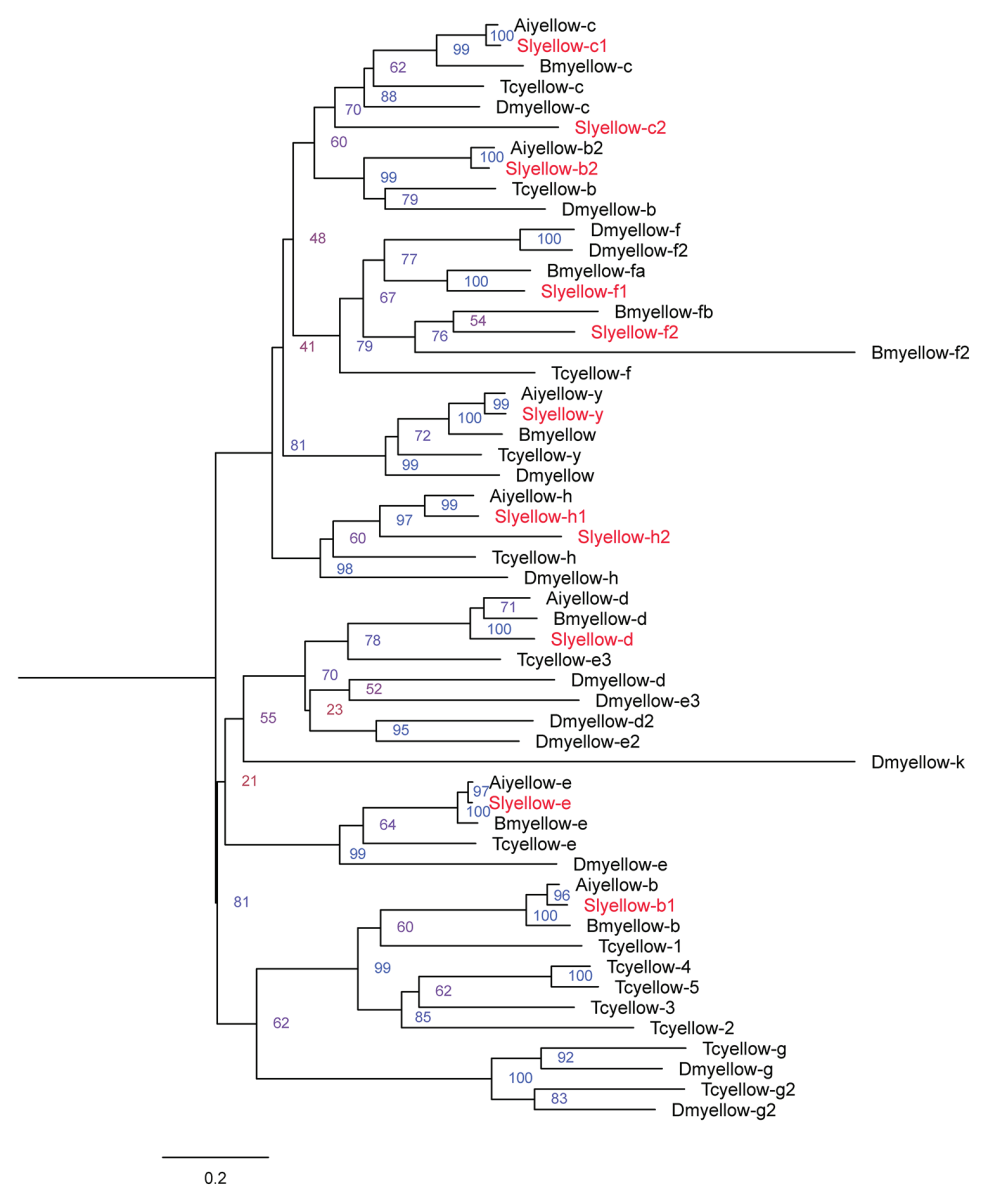
Phylogenetic analysis of the amino acid sequences of yellow proteins from *Spodoptera litura*, *Agrotis ipsilon*, *Bombyx mori*, *Drosophila melanogaster*, and *Tribolium castaneum*. The tree was constructed by neighbor joining method with a bootstrap of 1,000. Yellow proteins from *S. litura* are highlighted in red.

We obtained 11 Yellow proteins from NCBI database (Supplementary Table S2). The amino acid sequences of these 11 proteins together with those yellow proteins from *B. mori*, *T. castaneum*, *A. ipsilon*, and *D. melanogaster* were subjected to phylogenetic analysis using MEGA6.0. The 11 genes encoding *S. litura* yellow proteins were named according to their close relationships to characterized orthologs of other species (Figure 1).

Next, we validated the sequence of the *S. litura yellow-y* gene *via* end-to-end PCR. The complete ORF sequence of *yellow-y* consisted of 1,629 bp, encoding 542 amino acid residues. Amino acid sequence analysis showed that it contains a MRJP domain of about 300 amino acids, which is conserved in insect yellow proteins (Schmitzova et al., 1998; Supplementary Table S2). This sequence was used for further study.

### Stage and Tissue Expression Profiles of *yellow-y*

**Figure 2:**
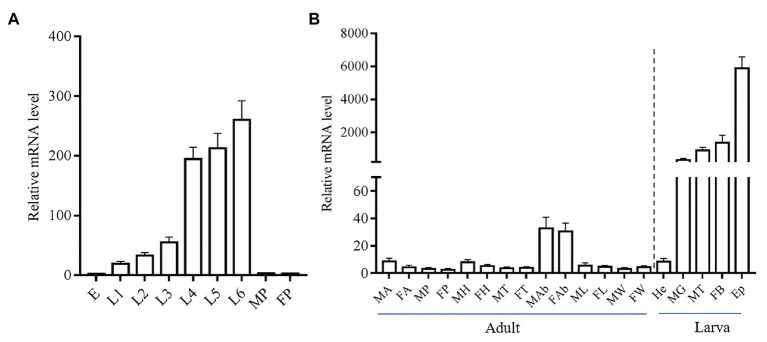
Relative expression levels of *yellow-y* mRNA at different development stages **(A)** and in different adult and larval tissues **(B)**. **(A)**. E, eggs; L1–L6, 1–6 instar larvae; MP, male pupae; and FP, female pupae. **(B)**. MA, male antennae; FA, female antennae; MP, male proboscis; FP, female proboscis; MH, male heads; FH, female heads; MT, male thoraces; FT, female thoraces; MAb, male abdomens; FAb, female abdomens; ML, male legs; FL, female legs; MW, male wings; FW, female wings; He, hemolymphs; MG, midguts; MT, malpighian tubes; FB, fat bodies; and Ep, epidermis. The mean value ± SE from three replicates are shown.

We investigated the stage and tissue expression pattern of the *yellow-y* by using real-time qPCR. The mRNA level of *yellow-y* increased gradually from egg stage to sixth instar larval stage, but was almost not detected in pupae stage (Figure 2A). As for the tissue expression profile in sixth instar larvae, *yellow-y* had the highest expression level in epidermis (Ep) followed by fat bodies, malpighian tubes (MT), and MG. The expression of *yellow-y* in larval hemolymphs and various adult tissues were very low (Figure 2B).

### Phenotypes Induced by CRISPR/Cas9 at Different Developmental Stages

**Figure 3:**
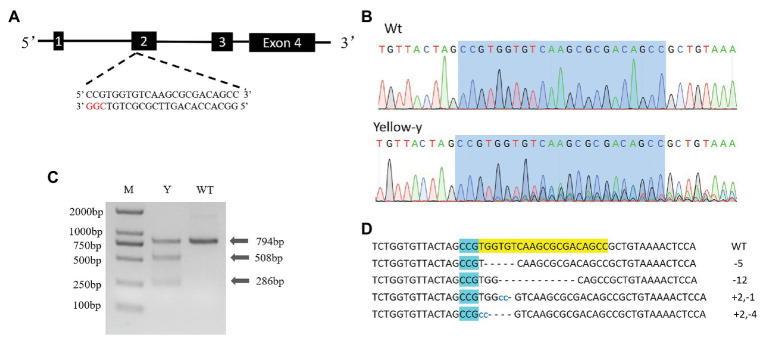
Clustered regularly interspaced short palindromic repeats/CRISPR-associated protein 9 (CRISPR/Cas9) directed mutagenesis of *yellow-y*. **(A)** The *yellow-y* gene has four exons. The sgRNA was designed in exon 2. The protospacer adjacent motif (PAM) sequence (CGG) is in red. **(B)** Representative sequencing chromatograms of PCR products from wild-type (WT; top) and G0 injected eggs (bottom) with the targeted site shown in blue shadow. **(C)** Cleavage events were detected by a T7E1 assay. The digestion products are indicated by arrows. Y, individual mutants; WT, wild type. **(D)** Mutant sequences determined by TA clone sequencing. The WT sequence is shown at the top with the PAM sequence in blue shadow. In mutant sequences, deletions are shown as dashes and insertions as blue lowercase. The change in length is marked at right of each sequence (+, insertion; −, deletion).

We used the CRISPR/Cas9 system to determine the function of the *yellow-y* gene in *S. litura*. To test whether the injection of sgRNA/Cas9 induced the mutation, we amplified and sequenced the targeted region of the *yellow-y* genomic sequence from the mixture of 20 G0 eggs. The chromatograms showed that insertion-deletion (indel) mutations were indeed induced in the G0 individuals by the injection (Figure 3B). Using the T7 Endonuclease 1 (T7E1) assay on PCR product from injected eggs, we were able to discern indel mutations based on the cleavage of heteroduplexes double-stranded DNA (Figure 3C). To further obtain the detailed information of the indel sequences, the PCR products of *yellow-y* were purified and subjected to the TA cloning, and positive clones were randomly selected for sequencing. Four out of the six successfully sequenced clones displayed different mutations, including two deletions and two mixed indel at the target site (Figure 3D).

**Figure 4:**
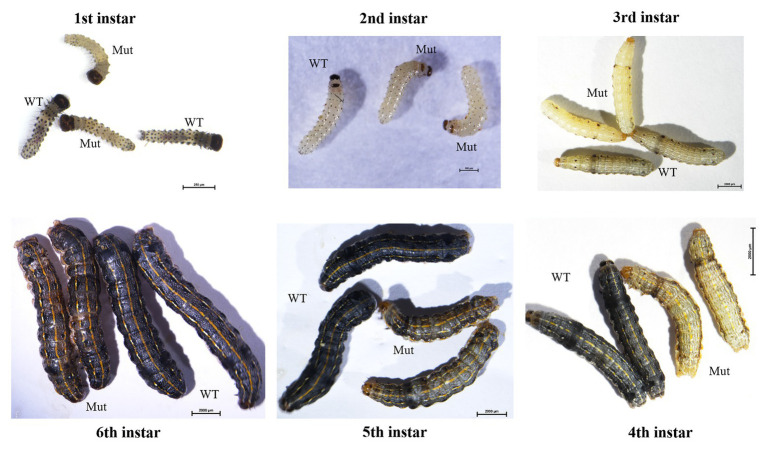
Phenotypes of *yellow-y* G0 mosaic mutants at six larval stages. The mutants show abnormal pigmentation at head capsule and body. WT, wild type; Mut, mutants.

**Figure 5:**
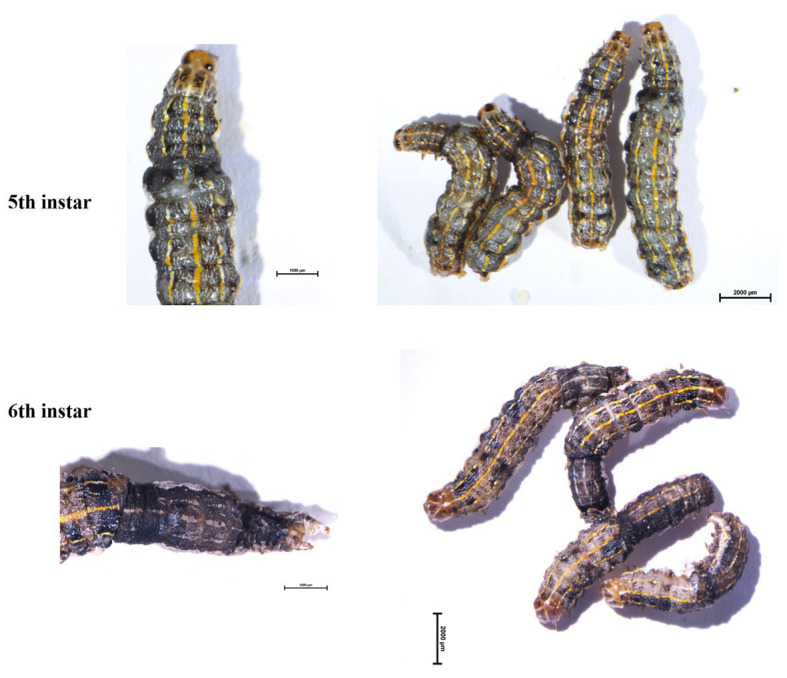
Phenotypes of G0 mosaic mutants at fifth instar and sixth instar larvae. The mutants show the abnormal body segmentation in fifth instar larvae and abnormal molting in sixth instar larvae.

**Figure 6:**
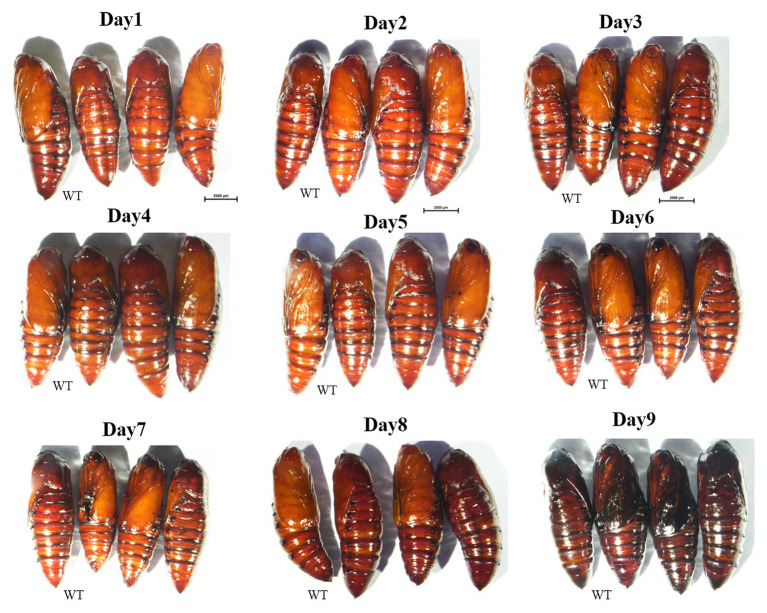
Phenotypes of *yellow-y* G0 mosaic mutants in pupal stage from day 1 to day 9. No significant differences in pigmentation were observed between mutants and WT ones. WT, wild type; Mut, mutants.

**Figure 7:**
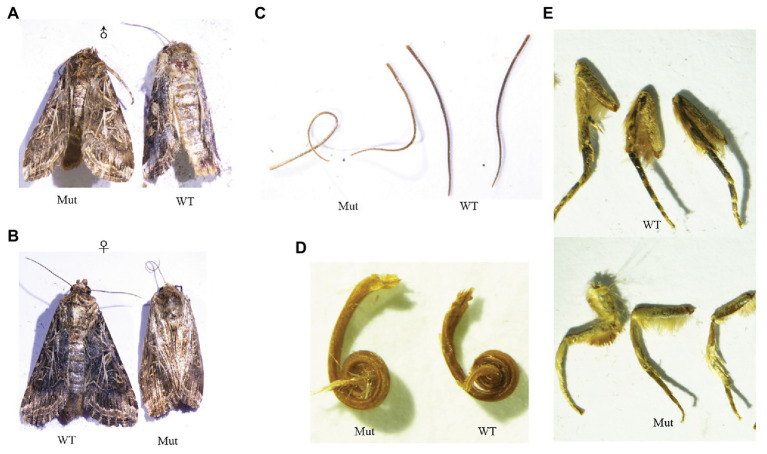
Phenotypes of *yellow-y* G0 mosaic mutants in adults. The mutants are yellower than wide type from the dorsal view of male **(A)** and female adults **(B)**, antennae **(C)** proboscis **(D)**, and legs **(E)**. WT, wild type; Mut, mutants.

**Figure 8:**
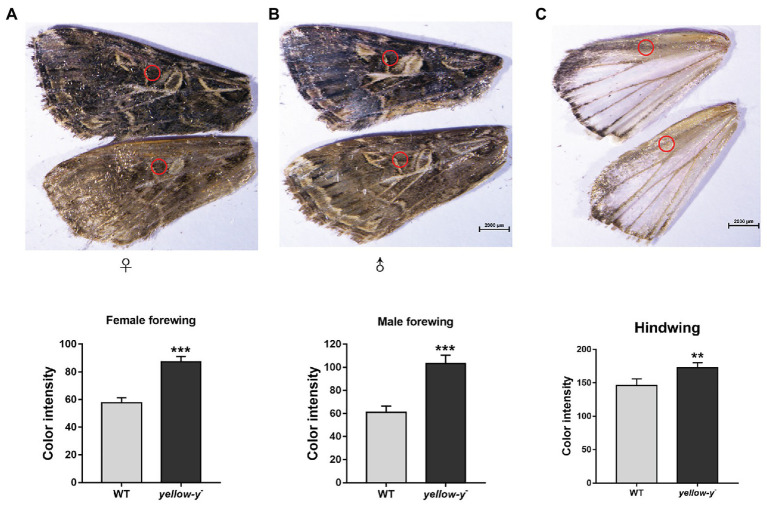
Phenotypes of yellow-y G0 mosaic mutants in wings. The yellow of pigment in equivalent regions of cuticles in female **(A)** and male forewing **(B)** as well as hindwing **(C)** from WT and mutant adults was measured using ImageJ software. The asterisk indicates a significant difference in color intensity of wing between WT and *yellow-y* ^−^ (*p* < 0.01, *t*-test).

After hatching, larvae of the Cas9/sgRNA-injected colony were raised and phenotyped at different developmental stages. We could clearly see that the color of both the head capsule and body wall turned yellow in larvae derived from the injected eggs, especially at first to fourth instar (Figure 4). The baenopoda and proleg of the larvae were also yellower than those of WT larvae (Supplementary Figure S1). Of the 53 G0 larvae, 11 individuals displayed partial body deformation at fifth instar stage, and 19 individuals could not molt normally at sixth instar stage (Figure 5; Table 1). However, we did not see any obvious color difference between G0 mutants (Mut) and the wild-type (WT) throughout the pupae stage, which indicated that *yellow-y* was not required for cuticle pigmentation at the stage (Figure 6). For adults, we found the truncus, wings, antenna, proboscis, and legs were yellower in the mutant insects (Figure 7). Further comparison showed that the color intensity of forewings and hindwings was markedly increased in the mutants (Figure 8).

**Table 1:** Mutagenesis number and rate of yellow-y gene were induced by Cas9/sgRNA.

	Injected eggs	Hatched larvae	mosaic mutants	deform larvae	Failure of larvae molt	Live pupae	Live adults
*Yellow-y*	200	80 (40.0)	53 (66.3)	11 (20.7)	19 (35.8)	18	15
ddH_2_O	200	145 (72.5)	–	–	–	115	102

To verify the agreement between the phenotype (yellow-like) mutants and the genotype mutants, genomic DNAs from fourth, fifth, and sixth instar larvae, male and female adult were respectively amplified and sequenced. The results showed that mutations were present at the target site of *yellow-y* in all yellow-like insects (Supplementary Figure S2), confirming the function of *yellow-y* in cuticle formation and coloration at larval and adult stages.

## Discussion

The *yellow* gene repertoires have been identified at genomic level in several genome sequenced insects, showing seven members in *B. mori* (Xia et al., 2006), 14 in *T. castaneum* (Arakane et al., 2010), 25 in *Nasonia vitripennis* (Werren et al., 2010), 14 in *D. melanogaster* (Drapeau, 2001), and seven in *A. ipsilon* (Chen et al., 2018). Here, in *S. litura*, there were 11 *yellow* genes, much more than that in *A. ipsilon*, also a Noctuidae species. The diversification in numbers of *yellow* genes in different insects might be attributed by evolutionary differentiation. Nonetheless, members of yellow gene family share the conserved MRJP domain, suggesting their similar function in biology processes. It is predicted that most of the *yellow* genes are related to melanic pigmentation of insect appendages and body parts (Wittkopp et al., 2002a,b). However, the exact role of each *yellow* gene in insects remains mostly a mystery, due to lacking effective technique for functional study in non-model insects.

In *Lepidoptera*, the role of *yellow-y* has been characterized to be involved in melanic pigmentation in *B. mori* (Xia et al., 2006; Futahashi et al., 2008) and *A. ipsilon* (Chen et al., 2018). However, the tissue expression pattern of *yellow-y* gene was not very consistent between the two lepidopteran insects. In *B. mori, yellow-y* was highly expressed in head, malpighian tubules, testis, and ovary by reverse transcription PCR, whereas in *A. ipsilon*, it was only highly expressed in head and Ep by qPCR. Here, we found that *yellow-y* in larval *S. litura*, similar to that in *A. ipsilon*, was highly expressed in epidermis, and the expression level increased gradually from first to sixth instar larvae, suggesting a role of *yellow-y* in cuticle pigmentation as reported in *A. ipsilon* (Chen et al., 2018). Besides, the *yellow-y* gene also had considerable expression level in fat bodies, MT, and MG of larval *S. litura*. Considering the importance of these organs, the yellow-y gene might be also involved in physiology related with fat bodies, MT, and MG. The present study was focused mainly on the morphological phenotypes, and more biochemical and physiological changes after the gene knockout need to be further investigated in the future study.

Our observation of phenotypes with *yellow-y* knockout individuals support the hypothesis that *yellow-y* is involved in the cuticle pigmentation in larvae but not in pupae, which accords with the findings in *A. ipsilon* mutants (Chen et al., 2018). However, CRISPR/Cas9-treated pupae exhibited a yellow color in *P. xylostella* (Wang et al., 2020). Besides, some larvae showed deformed body segmentation at fifth instar and abnormal molting at sixth instar in *S. litura*, which was not showed in *B. mori* (Futahashi et al., 2008), *A. ipsilon* (Chen et al., 2018), and *P. xylostella* (Wang et al., 2020). Similarly, in *T. castaneum*, knockdown of *yellow-y* expression by RNAi made the adults unable to shed the pupal cuticle properly and died entrapped in their pupal cuticle (Arakane et al., 2010). The detailed mechanisms underlying this abnormal larval development deserve to be further explored.

The *yellow-y* gene also plays a role in cuticle pigmentation in adults. In the present study, CRISPR/Cas9-treated adults exhibited a yellow color in body, wings, antennae, proboscis, and legs of adult *S. litura*, similar as the findings in *D. melanogaster* (Wittkopp et al., 2002a,b, 2009), *A. ipsilon* (Chen et al., 2018), and *P. xylostella* (Wang et al., 2020). However, there were also studies with coleopteran species showed different results. RNAi-induced *yellow-y* knockdowns in *T. castaneum* had normal cuticle pigmentation of adult body, although the black pigmentation of hindwing was specifically reduced (Arakane et al., 2010); in *T. molitor*, the knockdown of *yellow-y* expression by RNAi resulted in normal body color at day 1 after eclosion, but in lighter color at older ages (Mun et al., 2020). These discrepancies indicate insect *yellow-y* gene is complex in function among different insect orders, although some RNAi related factors (such as differences in gene knockdown efficiency, and day of observation of the phenotype) might also be the reasons.

In conclusion, the spatial and temporal expression profiles of *yellow-y* gene were determined in *S. litura*. Further functional study using the CRISPR/Cas9 system, demonstrated that *S. litura yellow-y* gene play important roles not only in cuticle pigmentation at both larval and adult stages, but also in segmentation and molting of the older larvae. In particular, the function of *yellow-y* gene in larva segmentation and molting is the first report in *Lepidoptera*. Our results provide new insights into the functions of the *yellow-y* gene, as well as a useful marker gene for genome editing based studies.

## Data Availability Statement

The original contributions presented in the study are included in the article/Supplementary Material, further inquiries can be directed to the corresponding author.

## Author Contributions

X-LL conceived and designed the experimental plan and wrote the manuscript. X-LL, W-KH, L-JZ, and Y-LY performed the experiment. X-LL, W-KH, and L-JZ processed and analyzed the experiment data. S-LD, QY, JZ, and Y-CP provided important suggestions to help to modify the manuscript. All authors contributed to the article and approved the submitted version.

### Conflict of Interest

The authors declare that the research was conducted in the absence of any commercial or financial relationships that could be construed as a potential conflict of interest.

## Footnotes

^1^https://www.ncbi.nlm.nih.gov/orffinder/

^2^http://www.ebi.ac.uk/interpro/search/sequence-search

^3^https://imagej.nih.gov/ij/index.html
