# Supplementary material for: Clustered Regularly Interspaced Short Palindromic Repeats/CRISPR-Associated Protein 9 Mediated Knockout Reveals Functions of the yellow-y Gene in Spodoptera litura
Source: Front Physiol. 2020 Dec 17;11:615391. doi: 10.3389/fphys.2020.615391 (PMC7839173; doi:10.3389/fphys.2020.615391)
Supplement: Supplementary file 1 [file Data_Sheet_1.docx]

**Supplementary data**

**Table S1.** Primers used in the study.

| Primer name | Sequence (5´- 3´) |
| --- | --- |
| End-to-end cDNA verification |  |
| Yellow-y-F | ATGTTATCATTTCAGTTCACTACCA |
| Yellow-y-R | TTAATGTTCGTATACTTCGTAATTACG |
| qPCR of tissue and stage expression |  |
| Yellow-y-qF | CCCAGTTTGCTGTCTCTACTC |
| Yellow-y-qR | GTTAGCACCTCGTTCACCTAC |
| GAPDH-qF | CGTGTTCCTGTTGCTAAC |
| GAPDH-qR | CTTGACCTTCTGCTTGATAG |
| EF1α-qF | ACGCTCCCGGACACAGAGAT |
| EF1α-qR | GCTCACGGGTCTGTCCGTTC |

**Table S2.** The gene family encoding yellow-like proteins in *S. litura*.

| name | cDNA (ORF) | Exons | amino acid | MRJP Domain (aa) | Genbank accession no. | |
| --- | --- | --- | --- | --- | --- | --- |
| yellow-b2 | 1377 | 3 | 458 | 118-403 | XP_022814035 |  |
| yellow-y | 1629 | 4 | 542 | 139-424 | XP_022829407 |  |
| yellow-b | 1350 | 1 | 449 | 125-403 | XP_022835357 |  |
| yellow-e | 1239 | 8 | 412 | 111-384 | XP_022834441 |  |
| yellow-c | 1227 | 8 | 426 | 119-407 | XP_022816607 |  |
| yellow-d | 1269 | 9 | 422 | 124-413 | XP_022834439 |  |
| yellow-c | 1266 | 9 | 421 | 117-404 | XP_022814199 |  |
| yellow-h | 1269 | 3 | 422 | 112-406 | XP_022835105 |  |
| yellow-h1 | 1269 | 4 | 422 | 121-408 | XP_022835108 |  |
| yellow-f | 1281 | 9 | 426 | 139-424 | XP_022831259 |  |
| yellow-fa | 1455 | 12 | 484 | 176-461 | XP_022831247 |  |

**Fig S1.**


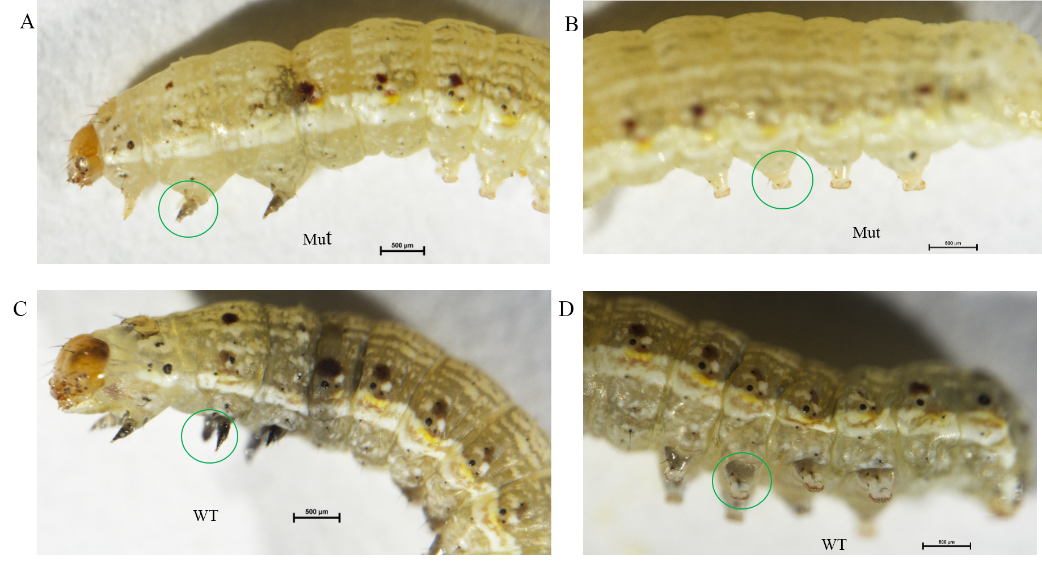


**Fig S1.** Phenotypes of G0 mosaic mutants at 3rd instar. The baenopoda (A, C) and proleg (B, D) were also observed yellower than WT. WT, wild type; Mut, mutants.

**Fig S2.**


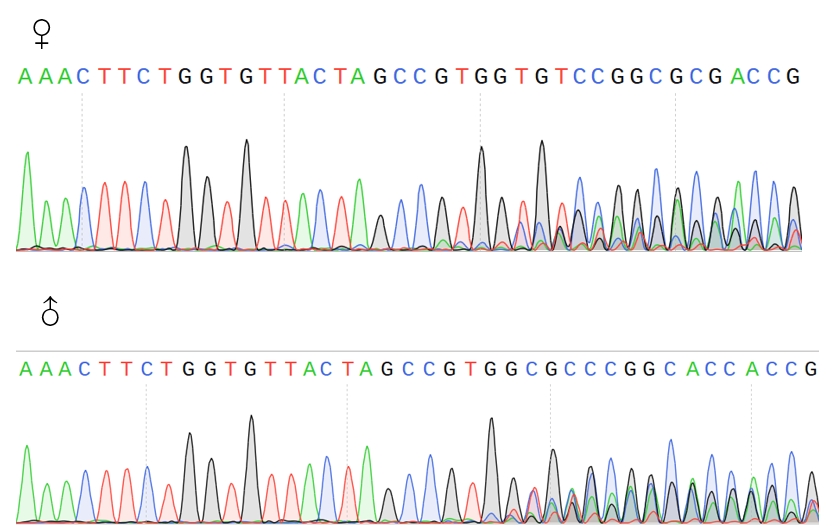


**Fig S2.** Representative sequencing chromatograms of PCR products from female (top) and male adult (bottom) in yellow color with the targeted site showed a stretch of double peaks, respectively.
